# Sonographic Assessment of the Effects of Mechanical Ventilation on Carotid Flow Time and Volume

**DOI:** 10.7759/cureus.20587

**Published:** 2021-12-21

**Authors:** Jessica I Schleifer, Lauren Ann J Selame, Jorge Short Apellaniz, Michael Loesche, Hamid Shokoohi, Carolyn Mehaffey, Andrew Liteplo

**Affiliations:** 1 Department of Anesthesia and Intensive Care Medicine, University Hospital Bonn, Bonn, DEU; 2 Emergency Department, Brigham and Women's Hospital, Harvard Medical School, Boston, USA; 3 Emergency Medicine, Hospital Fundacion Jimene, Madrid, ESP; 4 Emergency Department, Massachusetts General Hospital, Harvard Medical School, Boston, USA; 5 Anesthesiology Department, Massachusetts General Hospital, Harvard Medical School, Boston, USA

**Keywords:** critical care ultrasound, carotid ultrasound, mechanical ventilation, carotid blood flow, carotid flow time

## Abstract

Background

Corrected carotid flow time (CFTc) and carotid blood flow (CBF) are sonographic measurements used to assess fluid responsiveness in hypotension. We investigated the impacts of mechanical ventilation on CFTc and CBF.

Materials and methods

Normotensive patients undergoing cardiac surgery were prospectively enrolled. Carotid ultrasound (US) was performed pre and post-intubation. Post-intubation measurements took place after the initiation of mechanical ventilation. To measure CFTc and CBF, a sagittal carotid view was obtained with pulse wave-Doppler (maximum angle 60°). CFTc was calculated with the Bazett formula (CFTc = systolic time/√cycle time). CBF was calculated using CBF (mL/min) = area (cm ^2 ^) x time average mean velocity (TAMEAN) (cm/sec) x 60 (sec/min). The maximum carotid diameter was measured at the level of the thyroid.

Results

Twenty patients were enrolled. Mean CFTc pre-intubation was 328 ms (SD 43.9 ms) compared to CFTc post-intubation 336 ms (SD 36 ms). There was no significant difference between pre and post-intubation CFTc (mean differences=-0.008; t(19)=-0.71, p=.49). Mean CBF pre-intubation was 487 mL/min (SD 176 mL/min) compared to CBF post-intubation 447 mL/min (SD 187 mL/min). There was no significant difference between pre and post-intubation CBF (mean differences= 40; t(19)=1.24, p=.23).

Conclusions

In this study of normotensive patients, there were no detected differences in CFTc or CBF pre and post-intubation with mechanical ventilation.

## Introduction

Hypotension and respiratory failure characterize some of the most critically ill conditions treated in emergency departments and intensive care units. There has been much research focused on differentiating which hypotensive patients will respond to fluid boluses and which will not [1-4]. Both invasive and non-invasive means of predicting fluid responsiveness have been investigated [5-6]. There exist some publications on corrected carotid flow time (CFTc) and carotid blood flow (CBF) that suggest their usefulness in determining fluid status and responsiveness in non-ventilated patients [5,7-8]. A systematic review including studies of ventilated and non-ventilated patients supports the integration of carotid artery ultrasound (US) in hypotensive patients in order to predict fluid responsiveness [9]. However, there have not been studies focused on the effects of mechanical ventilation on CFTc and CBF.

While we know that mechanical ventilation can alter overall hemodynamics by increasing intrathoracic pressure and subsequently decreasing preload, we do not know if mechanical ventilation has important effects on carotid blood flow parameters. If mechanical ventilation does affect CFTc or CBF, this would be very relevant for critically ill patients who may be simultaneously hypotensive and require mechanical ventilation. In this study, we aimed to evaluate the impact of mechanical ventilation on carotid artery flow parameters in patients undergoing planned intubation in the operating room.

## Materials and methods

Study design and setting

This was a prospective observational study of a convenience sample of patients undergoing planned surgery in the cardiac operating room. The study was conducted between August 2019 and October 2019. The hospital institutional review board approved the study. Informed consent was obtained from all patients.

The pre-intubation US was performed with the spontaneously breathing patient in a supine position. After intubation but before the beginning of surgery, once a stable ventilator setting was in place, the post-intubation US was performed. Ventilator settings, including positive end-expiration pressure (PEEP), maximum pressure (Pmax), tidal volume, and respiratory rate, were recorded. Vital signs were measured at the time of US measurements. All data were collected and recorded using a standardized method. Our predictor variables were the change in CFTc and CBF pre and post-intubation.

Selection of participants

We enrolled a convenience sample of adult (≥18 years) patients scheduled for planned surgery with general anesthesia in the cardiac operating rooms. Enrollment was limited to those patients in whom US of the neck would not interfere with the planned surgery. Patients were excluded if they were <18 years of age, pregnant, did not have a regular cardiac rhythm, or had a history of advanced carotid artery disease, severe aortic stenosis, or aortic insufficiency.

All study staff were trained to assess CFTc and CBF using a standardized technique. Two emergency medicine US fellows experienced in the carotid US obtained all images.

Ultrasound measurements

A standardized scanning protocol was followed. The carotid artery was scanned in transverse and longitudinal orientations from the carotid bulb proximally toward the chest distally. Images were acquired with a portable US system (Mindray M9) using the linear-array (12-4 MHz) transducer with the “Carotid” preset (Mindray Medical Systems, Shenzhen, China).

**Figure 1:**
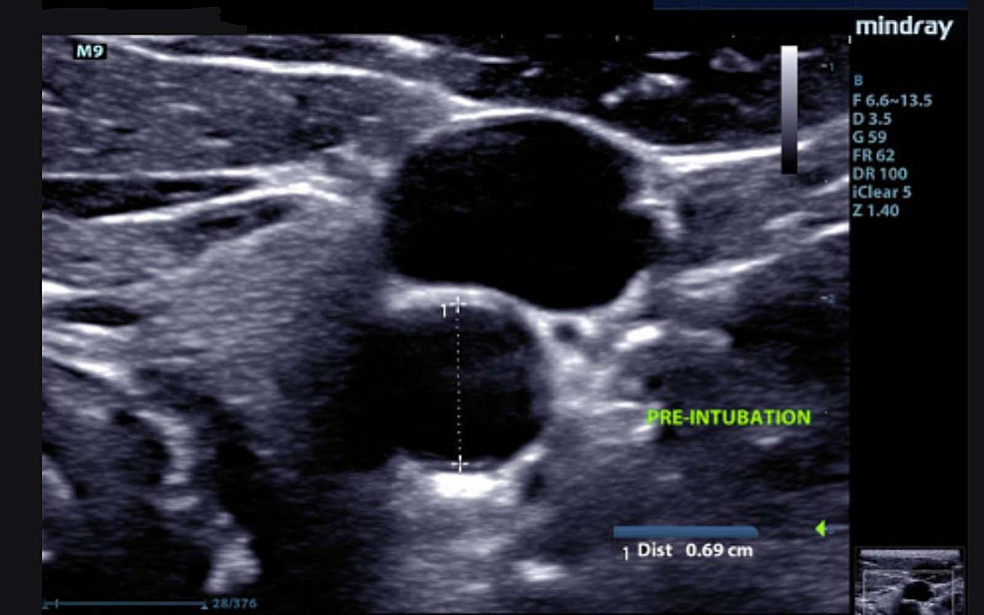
Carotid Artery Diameter The carotid artery vessel diameter was measured in transverse at the height of the thyroid gland or 3 cm below the carotid bulb if no thyroid gland were present. Pulse wave Doppler measurements were obtained at this same location. The diameter was transferred to the longitudinal image to allow the ultrasound machine to calculate the area and flow volume.

PW Doppler was used in a long-axis view of the vessel below the carotid bulb at the level of the thyroid or 3 cm below the carotid bulb if no thyroid was visualized. Transducer position, steering, and correction angle were optimized to the given anatomy to create an insonation angle <60°. The gate size was adjusted to cover the whole carotid artery, and the center of the gate was placed in the center of the vessel. The cross-sectional area of the vessel was calculated using the transverse maximal diameter at the height of the thyroid gland or 3 cm below the carotid bulb if no thyroid were present (Figure 1).

**Figure 2:**
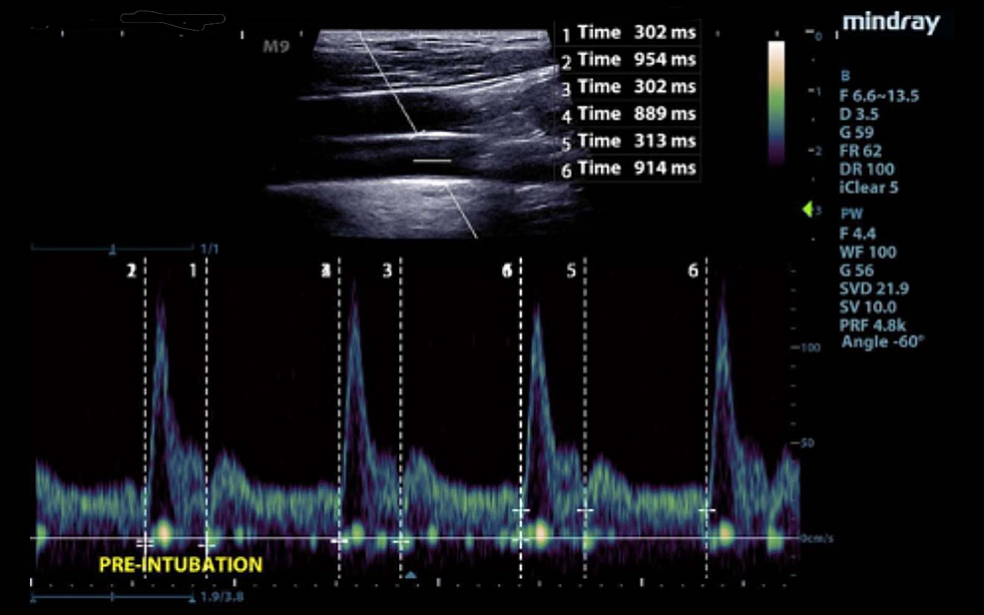
Flow Time and Cycle Time Pulse wave Doppler with measurement of flow time and cycle time over three cycles

For CFTc parameters, systolic time was measured from the beginning of the upstroke of the Doppler signal to the dicrotic notch (Figure 2).

Cycle time was measured from one upstroke to the next. CFTc was then corrected for HR using Bazett’s formula: CFTc = systolic time/√cycle time, with all times expressed in seconds. Three cycles were measured whenever possible, and the mean was used for calculations to improve precision.

**Figure 3:**
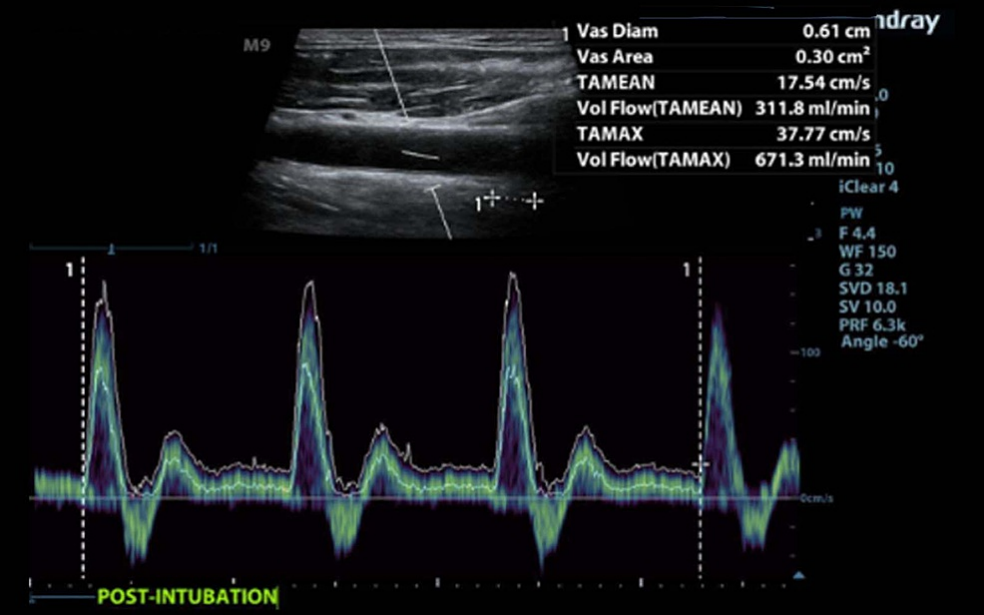
Flow Volume Pulse wave Doppler with measurement of flow volume, Time Average Mean Velocity (TAMEAN). The vascular diameter was measured in transverse at the same location as the doppler image. The diameter result was transferred to the longitudinal image so the ultrasound machine could calculate the area and the flow volume.

CBF was measured with the formula: CBF (mL/min) = Area (cm^2^) x time average mean velocity (TAMEAN) (cm/sec) x 60 (sec/min), where TAMEAN was the time average mean velocity obtained by an automatic pulse wave (PW) Doppler tracing (Figure 3).

Images were saved and measurements were made in real-time. All data were entered into REDCap (Research Electronic Data Capture), a secure Health Insurance Portability and Accountability Act-compliant web-based application hosted by our institution [10-11].

Statistical analysis

Cohort demographic and clinical data are reported in means with standard deviations for continuous variables and percentages for categorical variables. Paired sample t-tests were conducted to compare pre and post-intubation CFTc and CBF. Analysis of influencing factors on CBF and CFTc including PEEP, maximum pressure (Pmax), heart rate pre and post-intubation (HR-pre and -post), and mean arterial pressure pre and post-intubation (MAP-pre and -post) were conducted using Pearson’s product-moment correlations. The association between the previously described features and the pre and post-intubation differences were also conducted using Pearson’s product-moment correlations. All statistical analyses were performed in the R statistical programming environment [12].

## Results

Characteristics of study subjects

A convenience sample of 24 patients was enrolled; 20 patients completed the study and were used in the analysis. Patient characteristics are displayed in Table 1.

**Table 1:** Patient Characteristics (n=20) MAP: mean arterial pressure; PEEP: positive end-expiration pressure; HR: heart rate

	Mean	Standard Deviation (SD)
Age (years)	60	18
American Society of Anesthesiologists (ASA) score	3.3	0.7
HR pre-intubation (bpm)	72	15
Diastolic blood pressure pre-intubation (mmHg)	77	15
Systolic blood pressure pre-intubation (mmHg)	148	21.9
MAP pre-intubation (mmHg)	101	15.5
Intravenous fluids volume by time of second US (mL)	305	175
Tidal volume (mL)	446	55
PEEP (cm H2O)	5	1.4
Maximum pressure (cm H2O)	17.6	5.1
HR post-intubation (bpm)	65	15.2
Diastolic blood pressure post-intubation (mmHg)	69	14
Systolic blood pressure post-intubation (mmHg)	125	126
MAP post-intubation (mmHg)	86	16.5

Pre and post-intubation carotid parameters

**Figure 4:**
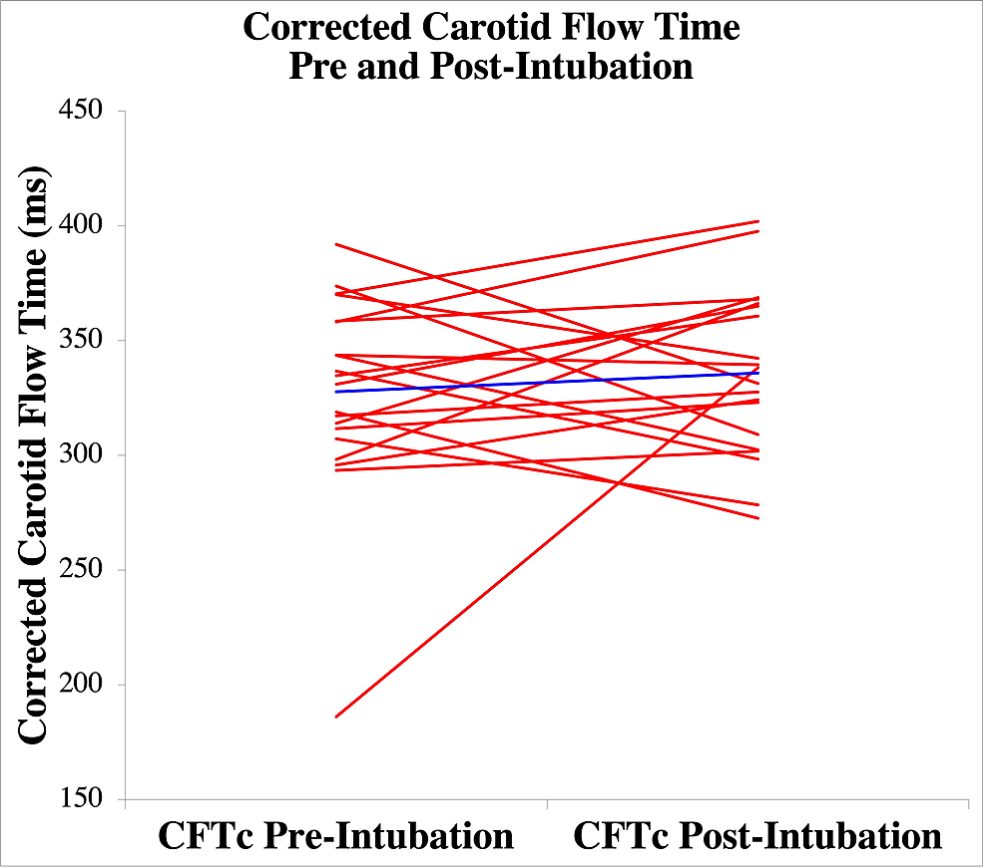
Corrected Carotid Flow Time Pre and post-intubation corrected carotid flow time (CFTc) is depicted for each subject with individual subjects represented by each red line and mean values represented by the blue line.

Mean CFTc pre-intubation was 328 ms (SD 43.9 ms) compared to CFTc post-intubation 336 ms (SD 36 ms). There was no significant difference between pre and post-intubation CFTc with mean differences = -0.008; t(19) = -0.71, p = .49. Based on our sample size, with a power of 80%, we were able to detect a difference in CFTc of 35 msec. Figure 4 visually depicts CFTc pre and post-intubation results for the group of patients, demonstrating overall similar measurements, aside from one outlier, before and after intubation.

**Figure 5:**
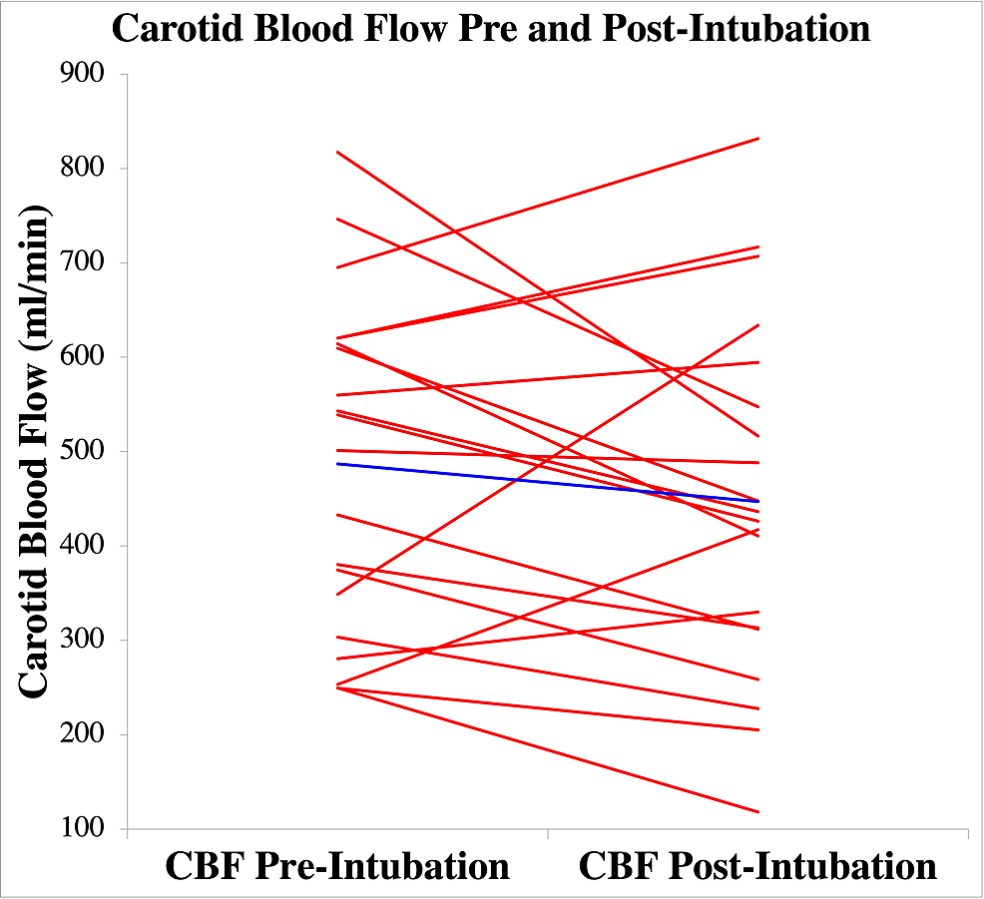
Carotid Blood Flow Pre and post-intubation carotid blood flow (CBF) is depicted for each subject with individual subjects represented by each red line and mean values represented by the blue line.

Mean CBF pre-intubation was 487 mL/min (SD 176 mL/min) compared to CBF post-intubation 447 mL/min (SD 187 mL/min). There was no significant difference between pre and post-intubation CBF with mean differences = 40; t(19) = 1.24, p = .23. Based on our sample size, with a power of 80%, we were able to detect a difference in CBF of 159.6 ml/min. Figure 5 visually depicts CBF pre and post-intubation results, depicting comparable pre and post-intubation CBF measurements for the group of patients.

Correlations with influencing factors

There was no correlation identified between the CBF or CFTc and PEEP, maximum pressure (Pmax), heart rate pre- and post-intubation (HR-pre and -post), and mean arterial pressure pre- and post-intubation (MAP-pre and -post). There was no correlation identified between the pre or post-intubation or the difference between the CBF or CFTc and PEEP, Pmax, HR-pre and -post, and MAP-pre and -post.

## Discussion

While we predicted that mechanical ventilation would have effects on carotid flow parameters, our results did not demonstrate this. Given known decreases in preload after initiation of mechanical ventilation and subsequent lowering of blood pressure, and known changes in carotid flow time with volume status changes, we anticipated both a decreased stroke volume and carotid blood flow [7,13-14].

In this cohort analysis, we did not find statistically significant changes in CFTc or CBF in response to mechanical ventilation as measured by US. Influencing factors of PEEP, Pmax, HR-post, and MAP-post did not show correlations with either CFTc or CBF. This suggests that for this group of patients, these parameters either do not affect CFTc and CBF, the sonographic signals were too noisy to detect differences if they do exist, or the sample was too small.

These results are reassuring, as they support the idea that mechanical ventilation does not have adverse effects on carotid artery flow for stable patients undergoing elective procedures in the operating room. However, as these were stable patients not requiring emergent intubation for respiratory failure or patients with hypotension, generalization of these findings to either hypotensive patients or those with respiratory failure may be limited. There have been small studies performed in critical care settings aimed to explore the relationship between a patient's internal jugular vein to common carotid artery diameter ratio (IJV/CCA) with the patient's central venous pressure (CVP) as a means to predict volume status through non-invasive means. While correlations were found between IJV/CCA and CVP in non-ventilated patients, there were no correlations found for ventilated patients [15-16]. Additional research is needed to better understand the effects of mechanical ventilation on carotid parameters for critically ill patients.

This study is limited primarily due to the single-center nature, small sample size, and convenience sample methodology. As this study was performed in a controlled setting in the operating room, the results have limited generalizability to other settings, including critically ill patients in the emergency department or intensive care unit. Preoperative patients are closely monitored by anesthesiologists and certified registered nurse anesthetics who respond nearly immediately to hemodynamic changes with vasoactive medications that could, in this study, dull the effects of mechanical ventilation on carotid artery flow parameters.

## Conclusions

In this study of normotensive patients undergoing mechanical ventilation, there were no detected differences in CFTc or CBF between pre and post-intubation with mechanical ventilation. Further research would be needed to investigate the generalizability of these findings. The impact of mechanical ventilation on critically ill patients remains unknown.
